# The Dental Cosmos

**Published:** 1872-01

**Authors:** 


### EDITORIAL DEPARTMENT.

---

*The Dental Cosmos.*—Prof. J. H. McQuillen, after a service of thirteen years, has closed his editorial connection with this magazine.

With professional engagements and collegiate duties occupying all the working hours of the day, it necessarily follows that magazine and other work must be attended to at times which the majority devote to social enjoyment; and only those engaged in such duties can estimate the sacrifice at which they are performed.

The honor of having maintained the *Dental Cosmos* in the front rank of dental literature, may be equally divided between Prof. McQuillen and its enterprising Publisher, and while we regret this change, we are pleased to learn that Prof. McQuillen's pen will yet be employed, as time and opportunity may offer, in contributing to the literature of the profession.

